# Female craniometrics support the ‘two-layer model’ of human dispersal in Eastern Eurasia

**DOI:** 10.1038/s41598-021-00295-6

**Published:** 2021-10-21

**Authors:** Hirofumi Matsumura, Guangmao Xie, Lan Cuong Nguyen, Tsunehiko Hanihara, Zhen Li, Khanh Trung Kien Nguyen, Xuan Tinh Ho, Thi Nga Nguyen, Shih-Chiang Huang, Hsiao-chun Hung

**Affiliations:** 1 School of Health Science, Sapporo Medical University, Sapporo, Hokkaido Japan; 2 Guangxi Institute of Cultural Relic Protection and Archaeology, Nanning, Guangxi China; 3 College of History, Culture and Tourism, Guangxi Normal University, Guilin, Guangxi China; 4 Institute of Archaeology, Vietnam Academy of Social Science, Hanoi, Vietnam; 5 Department of Anatomy, School of Medicine, Kitasato University, Sagamihara, Kanagawa Japan; 6 Southern Institute of Social Sciences, Vietnam Academy of Social Science, Ho Chi Minh City, Vietnam; 7 Department of Culture, Sports and Tourism of Quang Nam, Tam Kỳ, Quang Nam Vietnam; 8 Quang Nam Provincial Museum, Tam Kỳ, Quang Nam Vietnam; 9 Department of Anthropology, National Taiwan University, Taipei, Taiwan, ROC; 10 Department of Archaeology and Natural History, Australian National University, Canberra, Australia

**Keywords:** Evolution, Anthropology, Archaeology

## Abstract

This study reports a cranio-morphometric analysis of female human remains from seven archaeological sites in China, Vietnam and Taiwan that date between 16,000 and 5300 BP. The aim of the analysis is to test the “two-layer” model of human dispersal in eastern Eurasia, using previously unanalysed female remains to balance the large sample of previously-analysed males. The resulting craniometric data indicate that the examined specimens all belong to the “first layer” of dispersal, and share a common ancestor with recent Australian and Papuan populations, and the ancient Jomon people of Japan. The analysed specimens pre-date the expansion of agricultural populations of East/Northeast Asian origin—that is, the “second layer” of human dispersal proposed by the model. As a result of this study, the two-layer model, which has hitherto rested on evidence only from male skeletons, is now strongly supported by female-derived data. Further comparisons reveal that the people of the first layer were closer in terms of their facial morphology to modern Africans and Sri Lankan Veddah than to modern Asians and Europeans, suggesting that the Late Pleistocene through Middle Holocene hunter-gatherers examined in this study were direct descendants of the anatomically modern humans who first migrated out of Africa through southern Eurasia.

## Introduction

Understanding the origins of the peoples of East Asia, Southeast Asia, and Oceania is hindered by the complexities of historical migratory processes, both temporal and geographical, as well as the unquantifiable degree of genetic exchange that has occurred since the Late Pleistocene, or even earlier. Tracing the population history of this region ultimately reduces into two main issues^1^. The first concerns the genealogical relationship between contemporary populations and the early anatomically modern humans (AMH) who migrated initially from Africa to eastern Eurasia. The second issue concerns the impact of the human population spread, with rice and millet farming technology, from the Yellow and Yangtze River regions of China after 9000 years BP, leading ultimately to population dispersal across eastern Eurasia and eventually the Pacific Islands^2–5^. Current debate about whether or not this demographic dispersal was driven by agricultural expansion, and how it might have articulated with pre-existing hunter-gatherer populations in eastern Eurasia, can be enhanced by investigating the initial expansion of AMH during the Late Pleistocene.

Arguing against the two-layer model discussed in this paper, the “regional continuity” or “local evolution” model favors a scenario of multiregional evolution for modern humans, as represented by Turner’s Sundadont/Sinodont hypothesis^6^. This hypothesis assumes that the array of non-metric dental traits possessed by present-day Southeast Asians, that is, “Sundadont” traits in Turner’s terminology, are direct evidence for long-standing population continuity, uninterrupted by admixture with “Sinodont” peoples from the north. Multivariate craniometric analyses by others^7,8^ have also demonstrated close affinities between prehistoric and modern Southeast Asians, but they are mainly focused on Neolithic and early Metal Age samples from Thailand, Vietnam and Laos, hence they are not directly relevant for the issues discussed here.

In our previous study, analysis of cranio-morphometric patterns in eastern Eurasian and Sahul specimens (approximately 800 skeletons from late Paleolithic through Iron Age contexts) strongly supported the “two-layer model” of AMH dispersal in these regions^1^. According to this model, the “first layer”, the original Late Pleistocene AMH colonizing population, shared a direct ancestry with present-day Indigenous Australians and Papuans. In Southeast Asia, this first layer is represented primarily by Hoabinhian hunter-gatherers whose skeletal remains date between 12,000 and 4000 BP, before the expansion of Neolithic farmers. In our previous study, we regarded the agricultural populations who cultivated rice (*Oryza sativa*; and some also cultivated millets) as the “second layer”, who brought northeast Asian cranial features into Southeast Asia during the Neolithic period (from ca. 4000 BP).

Significantly, this two-layer model has hitherto been based only on male data sets. Male skulls were used for a variety of reasons, especially that morphological divergence is greater among male than female crania owing to greater male cranial size and robustness. Additionally, there are more male than female archaeological specimens available for study.

**Figure 1:**
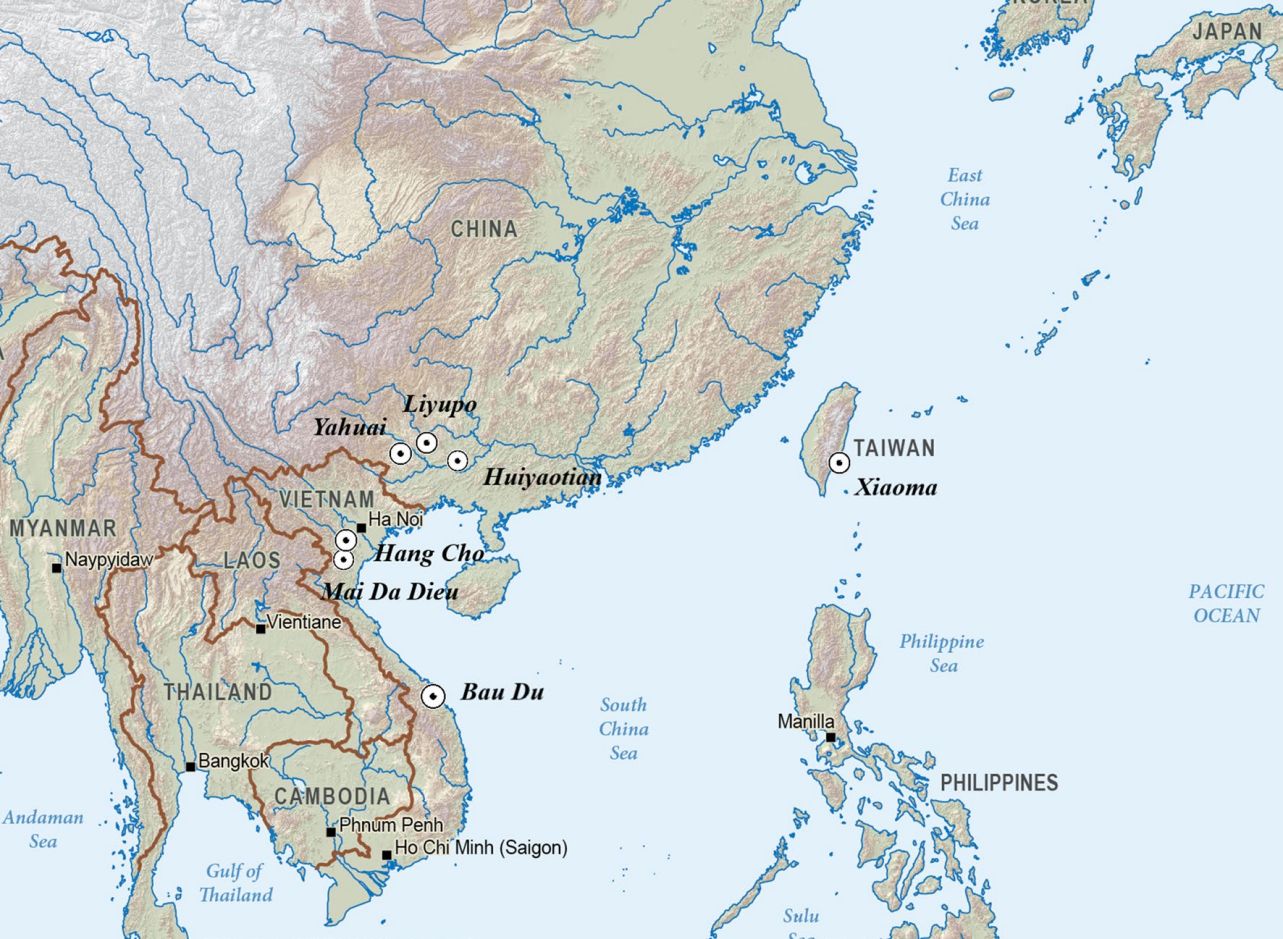
Locality map of the seven focal sites (Map generated by H.C. Hung from ANU CartoGIS CAP 00-210 under a CC BY license, with permission from CartoGIS Services, ANU Scholarly Information Services, The Australian National University, 2021).

Accordingly, our new study examines female skeletons from seven Late Pleistocene through Middle Holocene archaeological sites in China, Vietnam and Taiwan (Fig. 1: site location map). The oldest comes from Yahuai Cave (n = 1) in Guangxi Province, southern China. Nine more pre-farming individuals come from Huiyaotian (n = 6) and Liyupo (n = 3) in Guangxi Province, southern China. A further three come from Hang Cho (n = 1), Mai Da Dieu (n = 1), and Bau Du (n = 1) in Vietnam. One comes from Xiaoma Cave (n = 1) in southeastern Taiwan.

In the context of the two-layer model, these analyzed specimens are all presumed to have been members of the first layer of human dispersal. Our study compares the craniometric features of the aforementioned female specimens with those of contemporary populations in Africa, Eurasia, and Melanesia. Although many other female skeletons have been reported from Late Paleolithic, Neolithic and Iron Age sites across the region, the lack of standardization in the recorded measurements and the lack of published data make it difficult to include them. Instead, we use comparative data from modern populations measured according to our standard protocols.

### The study sites

**Figure 2:**
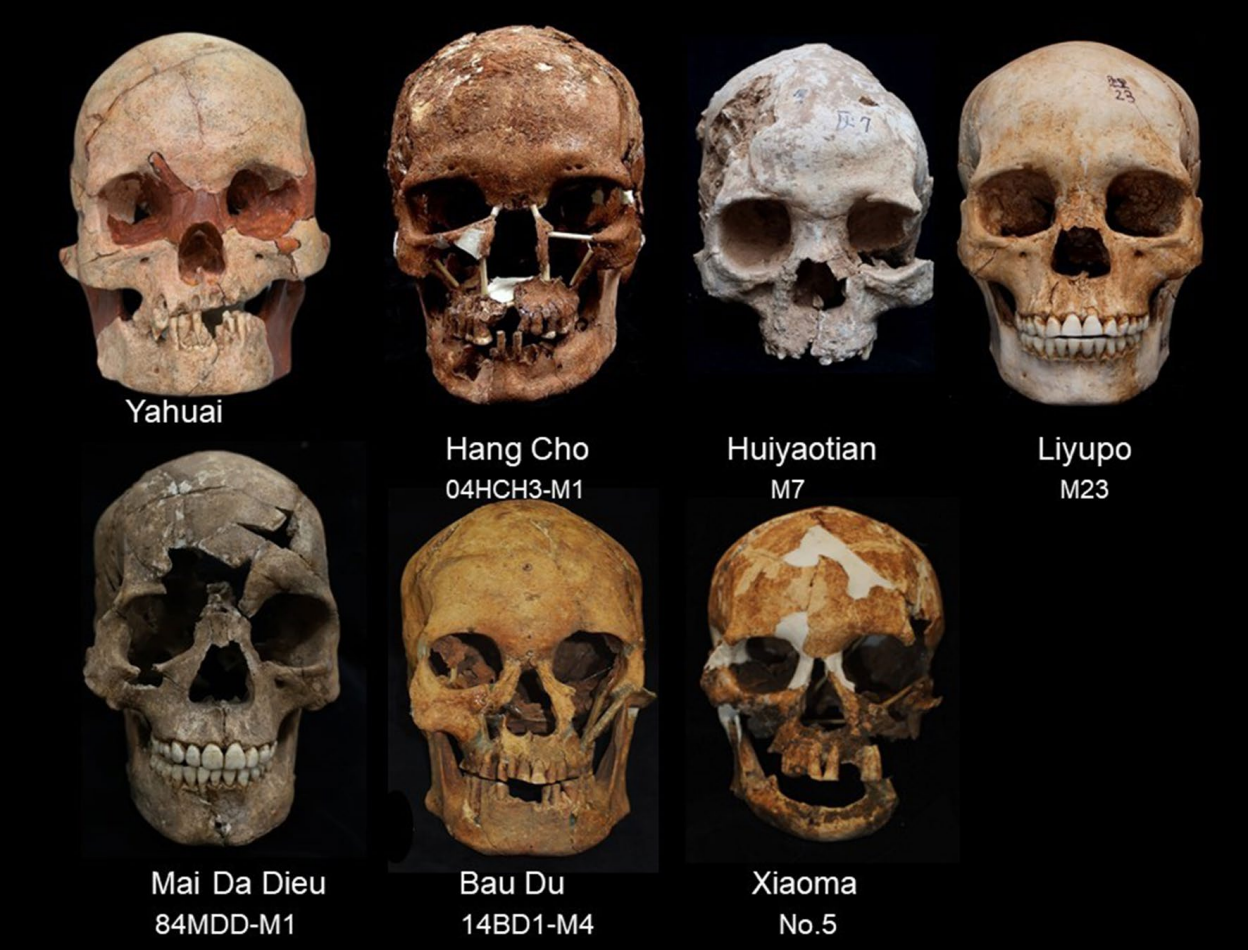
Frontal profiles of the seven analysed female crania.

Table 1 presents details of the analysed remains and archaeological sites, including pertinent radiocarbon dates. Figure 2 exhibits the analysed cranial specimens unearthed from these focal sites. Sex was determined from pelvic and cranial morphology, and other post-cranial markers^9^. Sex determinations for new individuals reported in this study are described, otherwise details can be found in the cited publications.

**Table 1:** AMS radiocarbon dating of human remains in this study.

Site and locality	Burial or layer number	Calibrated date range	Material dated	Laboratory code	Citation
Yahuai Cave, Long’an County, Guangxi, China	Layers 6, 7	16,355–16,086 BP	Charcoal	BA160129 BETA444301 BA160130	Wu et al.^10^
Huiyaotian Shell Midden, Nanning City, Guangxi, China	Layers 3–5	9030–8315 BP	Charred Canarium seeds, human tooth	BETA429237 BETA429238 BETA429239	Matsumura et al.^12^
Liyupo Site, Long’an County, Guangxi, China	Layers 2, 3; burial M12, M35	8025–6741 BP	Charred Canarium seeds, human tooth and bone, charcoal	IAAA143260 BETA429240 BETA429241 BETA429242	Matsumura et al.^12^
Hang Cho Cave, Luong Son District, Hoa Binh Province, Vietnam	04HcH3-M1	10,750–10,150 BP	Human tooth	No lab number; sample submitted by M. Yoneda	Matsumura et al.^15^
Mai Da Dieu Cave, Ba Thuoc District, Thanh Hoa Province, Vietnam	84MDD-M1	8200–7970 BP	Charcoal	Bln3540 Bln3541	Truong and Phong^19^
Bau Du Shell Midden, Nui Thang District, Quang Nam Province, Vietnam	14BD1-M4	5600–5270 BP; 5390–5270 BP	Charcoal	ANU54809–54814 (six samples) Bln3040	Nguyen^20^
Xiaoma Cave, Taidong County, Taiwan	C5	6189–5920 BP; 5996–5725 BP	Marine shells	No lab numbers; samples submitted by S.C. Huang	Huang and Chen^21^

### Yahuai Cave, Guangxi Province, China

**Figure 3:**
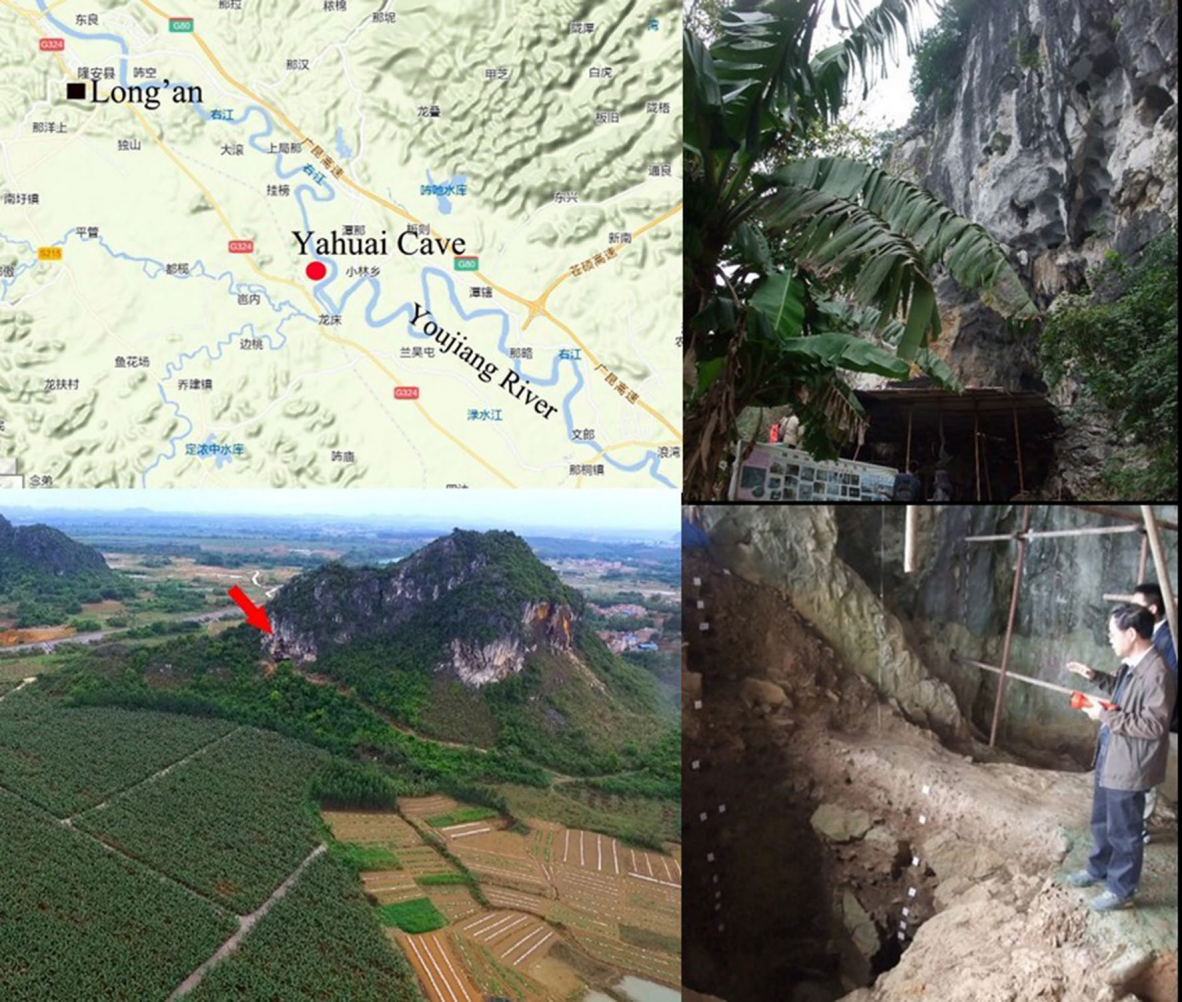
Map and views of Yahuai Cave, Guangxi, China (map generated by G. Xie from https://map.bmcx.com/, under a CC BY license).

Yahuai Cave is located in Long’an County, Guangxi Zhuang Autonomous Region, South China (107° 45′ 35″ E, 23° 6′ 25″ N), 100 km northwest of Nanning City (Fig. 3). The main rockshelter and inner cave enclose a total area of more than 100 m^2^. Three seasons of excavation were conducted between 2015 and 2018 by an archaeological team led by co-author Xie from the Guangxi Institute of Cultural Relic Protection and Archaeology^10^. The excavation was divided into four areas (A, B, C, and D), with a total exposure of 50 m^2^. Total depths reached 7.5 m at the rock floor in Area A, but cultural deposits are up to 5 m thick, most dating from 44,000 through 16,000 BP by AMS 14C dating.

A complete human skull with a partial mandible was discovered beneath layer 6 in Area B, and dated by three associated charcoal samples to 16,000 BP^10^ (Table 1). As the post-cranial skeleton was not exposed, sex determination is based on the skull. The perpendicular and elevated frontal bone, small mastoid processes, and the smoothness of the occipital muscle attachment area are all female characteristics.

Two hearths were found in the Pleistocene deposits at Yahuai. Tens of thousands of stone artifacts, some shell and bone tools, and an abundance of animal and plant remains (including wild rice phytoliths) were recovered. The lithic assemblage is dominated by small amorphous flakes and pebble tools of quartzite, sandstone, flint, tektite and quartz crystal^11^.

### Other comparative hunter-gatherer sites

#### Huiyaotian Shell Midden, Guangxi Province, China

The well-preserved Huiyaotian Shell Midden is located on the first terrace of the Yongning River in Qingxiu District, Nanning City, Guangxi. The site was discovered in 1973 and first excavated by a joint archaeological team from the Guangxi Institute of Cultural Relics and Archaeology (currently the Guangxi Institute of Cultural Relic Protection and Archaeology) and the Nanning City Museum in 2006. Co-authors Li, Matsumura, Hung and L. C. Nguyen then conducted further research in 2012^12^. The thickness of the cultural component is about 60–120 cm, in a matrix primarily composed of river shells. Three samples, including two charred *Canarium* sp. seed fragments and one human tooth, produced AMS C14 dates of 9000–8300 BP (Table 1). The assemblage belongs to the Dingsishan Cultural Phase in the Yongning River Basin, which pre-dated the arrival of rice-farming economies from the Middle-Lower Yangtze Basin^3,5^. In total, 64 inhumations were recovered, including flexed, squatting and some dismembered burials, all without grave goods. The artifacts recovered in association with the human remains included polished stone axes and adzes; a bone assemblage of adzes, arrowheads, awls, needles and fish-hooks; shell tools; and sherds of cord-marked pottery.

#### Liyupo, Guangxi Province, China

The Liyupo open site is located in Jianan Village, Dingdang Town, Long’an County, Guangxi. Surrounded by karst limestone hills, it consists of a 32 × 20 m soil mound. Liyupo was excavated in 2009 by the Guangxi Institute of Cultural Relics and Archaeology (currently the Guangxi Institute of Cultural Relic Protection and Archaeology), Nanning City Museum, and the Cultural Relic Management Institute of Long’an County. Since 2012, Matsumura, Hung, and L. C. Nguyen have collaborated with Li on the interpretation of the Liyupo bio-anthropological and archaeological materials^12^.

The excavation area in 2009 included three trenches totalling 26 m^2^. The deposit contained shells and artifacts, including polished stone axes and adzes, grindstones, small bone awls, and shell shovel-like artifacts. AMS C14 dates on charred *Canarium* sp. seeds, charcoal, and human bones and a tooth give a chronology of c. 8000–6700 BP (Table 1). In total, 40 flexed skeletons were unearthed, many deliberately protected under large stones^12^.

#### Hang Cho Cave, Hoa Binh Province, Vietnam

Hang Cho is a limestone cave site located near Cao Ram Village, Luong Son District, Hoa Binh Province, northern Vietnam. French archaeologist Madeleine Colani led the primary excavation in l926 and 1932, uncovering Hoabinhian stone tools^13^. Further investigation occurred by the Vietnamese Institute of Archaeology in l997–1998. In 2003, a research team from Hanoi National University and Seoul National University obtained freshwater shell AMS C14 dates of c. 19,500 and c. 8400 BP^14^.

In 2004, another multinational excavation project including Matsumura and L. C. Nguyen uncovered a flexed human skeleton (04HCH3M1) at the cave entrance^15^. Its pelvis has an obtuse-angled greater sciatic notch that suggests a female. Direct AMS radiocarbon analysis of a single tooth dates the skeleton to approximately 11,000 BP (Table 1). A total of 1523 Hoabinhian lithics were found in stratigraphic association with the skeleton, including a few edge-ground axes as well as pebble and flake tools.

#### Mai Da Dieu Cave, Thanh Hoa Province, Vietnam

Located near Ha Trung Village, Ba Thuoc District, Thanh Hoa Province, northern Vietnam, Mai Da Dieu was excavated by the Institute of Archaeology and the Thanh Hoa Provincial Museum between 1984 and 1989. L. C. Nguyen participated in the excavation at this time^16–18^. The site contains a long cultural sequence with C14 dates ranging between 19,700 and 3500 BP. Twenty six human burials have been recovered from the site, among them a young adult (84MDD1) found in well-preserved condition 0.7 m below the surface. The pelvis of this individual has an obtuse-angled greater sciatic notch, suggesting a female. Indirect C14 dating on charcoal indicates an age of c. 8000 BP for this individual, within a Hoabinhian archaeological context (Table 1).

#### Bau Du Shell Midden, Quang Nam Province, Vietnam

Bau Du is a shell midden located in Phu Trung Village, Nui Thanh District, Quang Nam Province, central Vietnam. The site was excavated initially in 1983–1984 by the Vietnam History Museum in Hanoi and the Quang Nam Provincial Museum^20^. In 2017, an international team including co-authors Matsumura, Hung, K.T.K. Nguyen, L.C. Nguyen, T.N. Nguyen, and several others (including Marc Oxenham, Ngoc Kinh Dang, Nguyen Kim Dung, and Hoang Bach Linh Nguyen) conducted another joint excavation on the site, with detailed results yet to be published. AMS analysis of 6 charcoal samples from this shell midden dates it to 5590–5270 BP (Table 1).

Bau Du produced pebble and flake tools, but no polished tools and no pottery. Several flexed human skeletons were found sealed in pits beneath the shell midden. This study examines the nearly complete skull of one of them (14BDHIL5M4) discovered in 2014^20^. The sex assignment as female is based on its obtuse-angled greater sciatic notch.

#### Xiaoma Cave, Taidong, Taiwan

Xiaoma is a limestone cave located in Chenggong Town, Taidong County, southeast Taiwan. The site was excavated in 1988–1990 by Huang and the National Taiwan University archeological team^21–23^. The excavation uncovered a flake tool assemblage, probably related to the late Changbin (Preceramic) cultural phase of east coastal Taiwan, and a burial pit dated on marine shell to c. 6200–5700 BP (Table 1). The Xiaoma human burial is the only known pre-Neolithic burial from the main island of Taiwan, and it was placed in a squatting position. Following its excavation in 1988, it was stored, without cleaning or reconstruction until this study, in the Anthropology Museum of National Taiwan University.

The short stature, short limbs, and small cranial size of the Xiaoma individual indicate a close affinity to Negrito (Ayta) groups in the Philippines. Although the Austronesian-language oral traditions of Formosan populations contain references to the existence of small-statured and dark-skinned people on the island at some time in the past^24^, this reference never has been confirmed by skeletal data, until now. Therefore, Hung, Matsumura, and L. C. Nguyen initiated a joint project on this skeleton in 2016, resulting in the reconstructed cranium presented here. Although the innominate bone of this individual is too damaged for sex determination, it is assumed to be female because of its gracile cranium, small mastoid processes, smooth occipital muscle attachment area, perpendicular and elevated frontal bone, and the smooth contour of its mandibular base.

## Results

### Craniometric affinities within eastern Eurasia and Melanesia

**Figure 4:**
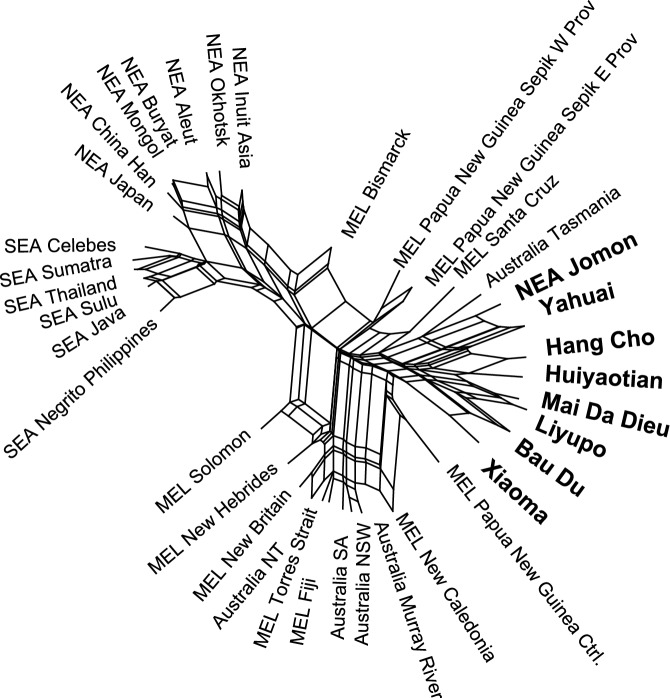
A Neighbor Net Tree generated by the distances of Q-mode correlation coefficients based on female cranial morphometric data from the seven study sites and samples from eastern Eurasia and Melanesia (NEA = Northeast Asia, SEA = Southeast Asia, MEL = Melanesia including Papua).

Our study commenced with an analysis of the female skulls just listed, together with others from mid-Holocene burials in Jomon Japan, in order to reveal cranial affinities within this population assemblage. Figure 4 depicts a tree diagram based on the Neighbor Net analysis of Q-mode correlation coefficients using 13 cranio-morphometric datasets (Tables S1 and S2). The diagram exhibits two major clusters. The cluster on the upper left comprises Northeast and Eastern Asians (NEA) as well as present-day Southeast Asians (SEA). The cluster on the lower right comprises Indigenous Australians and Melanesians (MEL, including Papuans).

### Craniometric affinities across Eurasia, Melanesia and Africa

**Figure 5:**
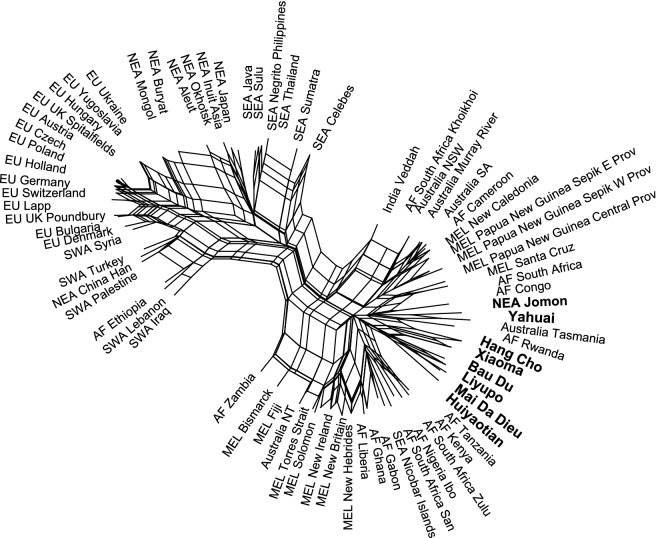
A Neighbor Net Tree generated by the distances of Q-mode correlation coefficients based on female cranial morphometric data from across Eurasia, Melanesia and Africa (NEA = Northeast Asia, SEA = Southeast Asia, SWA = Southwest Asia, EU = Europe, MEL = Melanesia including Papua, AF = Africa).

As Fig. 5 shows, analysis using a much larger data-set encompassing western Eurasian and African populations (Tables S3 and S4) produced a similar recognition of two major clusters. The mega-cluster on the upper left consists of four sub-clusters: Northeast Asians (NEA), Southeast Asians (SEA), Southwest Asians (SWA), and Europeans (EU). The mega-cluster on the right comprises Melanesians (MEL, including Papuans), Australians, South Asians (SA), Africans (AF), and the seven ancient samples discussed above.

The main observation to be drawn from this distribution is that the Australo-Papuan, South Asian Veddah, Nicobar Island and ancient Jomon samples, plus the seven ancient samples analysed in this study, have a closer affinity to African samples than they do to any other Eurasian samples included in this study.

### Principal component analysis

The PCA results using the mean data-sets of 13 standardized cranial measurements taken from 67 modern population samples are given in Table S5, with their eigenvectors, eigenvalues and loadings. Table S6 provides the PCA scores for all comparative population samples, including the focal prehistoric series. The loading values are of the first five components, the eigenvalues of which are greater than 1, and the cumulative contribution is 85.64%.

**Figure 6:**
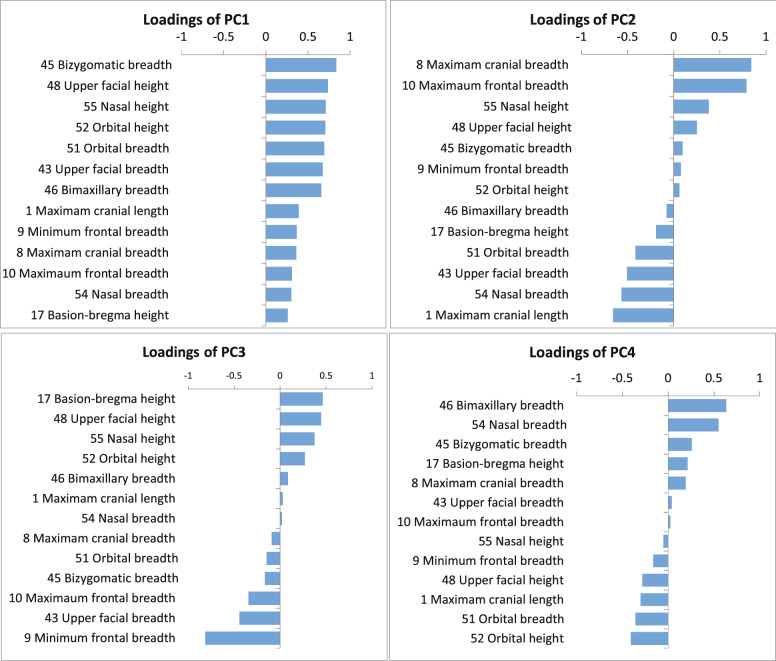
Loadings of the first four components of the Principal Components Analysis (PCA) applied to the 13 mean cranial metric data-sets for 67 female comparative modern population samples (females).

Figure 6 represents the loading values of the 13 cranial measurements for the first four principal components whose contribution rate is greater than 10%. The first component (PC 1) reflects the overall cranial size because all measurements almost equally indicate positive loading values.

In the second PCA component (PC 2), the cranial breadth is fairly positive, while the length of the cranial vault and the breadth of the facial segments are negative, thereby suggesting that the cranium with lower PC scores will be characterized by a broader face and longer head. The third component (PC 3) exhibits relatively high positive loadings for the facial and nasal height against faintly negative loadings of breadth of overall segments. A skull with a lower negative score in PC 3 will possess a broad lower face. Meanwhile, the loadings of PC 4 tend to be positive for bimaxillary and nasal breadth and negative for cranial vault length and orbital size. Accordingly, specimens with high scores will be characterized by a wide nasal opening against orbital size.

**Figure 7:**
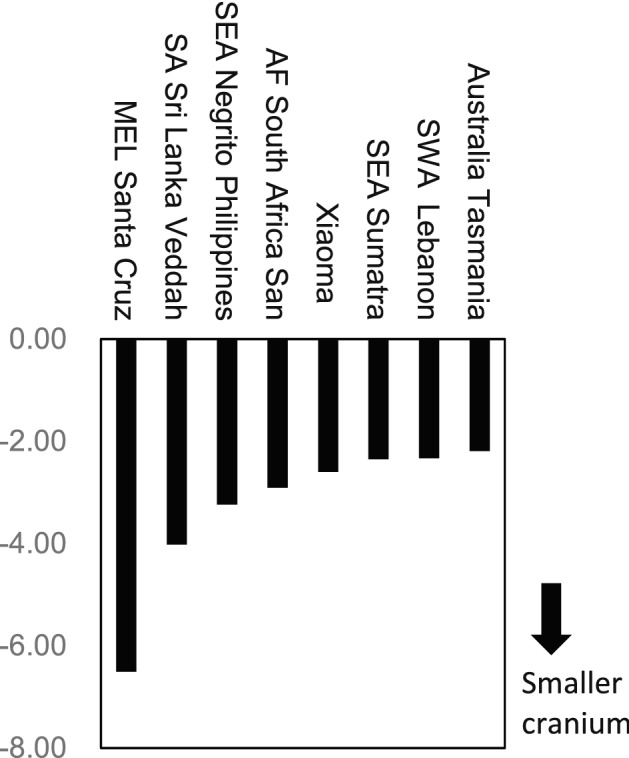
Female population samples with the top eight negative scores in the first component (PC 1) of PCA.

Figure 7 presents comparative samples, with the most negative scores in PC 1 calculated using the 13 cranial measurements. The cranial sizes of the Santa Cruz Melanesians, Veddah in Sri Lanka, Negrito in the Philippines, and San in South Africa are small, in accordance with their small body sizes—known features of these populations. Notably, the small size of the Xiaoma crania from Taiwan is comparable with these samples.

**Figure 8:**
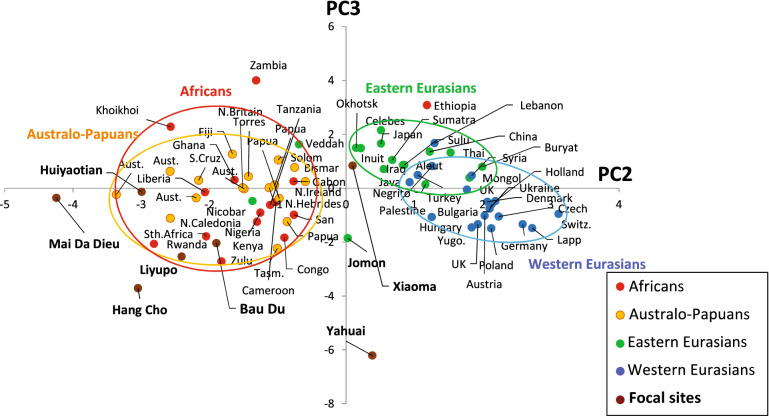
Two dimensional plots of PCA scores in the second and third components of the comparative female population samples.

Figure 8 plots the scores of PC 2 and PC 3 in a two-dimensional scheme. Northeast/Southeast Asians and Europeans are scattered on the right side, while Africans and Australo-Papuans—possessing a broader face and longer head—are clustered on the left side. With the exception of Xiaoma and Yahuai, the five focal specimens are fairly close to the Australo-Papuan and African assemblage in the lower left side of Fig. 8, illustrating an overall tendency toward relatively lower, broad faces with a long cranial vault. In this profile, Xiaoma as well as Jomon are intermediate, while Yahuai is particularly remarkable in possessing a broad lower face.

In terms of un-schematized PC 4, as given in Table S6, the seven focal series indicate high positive scores, especially for Xiaoma and Bau Du, characterizing a wide nasal opening against orbital size.

## Discussion

As mentioned, in a previous study on the dispersal process of AMH in eastern Eurasia, we proposed the two-layer model based on cranial data-sets of male specimens^1^. We proposed that the Late Pleistocene AMH colonizers (first layer) in southern China and Southeast Asia—akin to the ancestors of current Indigenous Australo-Papuans—were encountered during the Holocene by a second layer of migrants from northern Eurasia who possessed morphological characteristics adapted to a cold climate. The dispersal of this second layer of human population involved large scale demic expansion of agriculturists from the Neolithic period onwards.

This two-layer model of population history has been strongly supported by recent genome-wide ancient DNA analyses in East/Southeast Asia^25–28^. Indeed, these genomic studies suggest that the first layer, which equated archaeologically with the Hoabinhian in Mainland Southeast Asia, shared genetic resources with the ancient Jomon people of Japan^26^, as documented independently by the morphometric cranial affinities between these two populations that we have discussed above.

Against the two-layer model, the “regional continuity” Sundadont/Sinodont model proposes that modern Northeast Asians derive from Southeast Asian migrants, the reverse of what is suggested by us. The regional continuity model is not supported by our previous dental^29^ or male craniometric data^1^, or by our current study based on female craniometric data. Additionally, it is not corroborated by recent genetic studies^30^.

Our study shows that pre-Neolithic female hunter-gatherers from our sample of seven sites in southern China, Taiwan and Vietnam, as well as the Holocene Jomon population of Japan, all belonged to the first layer within our two-layer model. Our findings demonstrate a close cranial affinity between the analysed specimens and recent Indigenous Australians and Papuans. In terms of the Huiyaotian and Liyupo samples, the clustering pattern generated by female craniometric data is consistent with that based on male data in previous studies^1,12^, further underscoring the significance of the cranial affinity.

A comparison of the specimens that we have analysed with a larger population that extends as far as western Eurasia and Africa produces a more comprehensive interpretation. The pre-Neolithic skulls and recent Australo-Papuan samples exhibit a closer resemblance to African samples than do present-day Eurasian samples. In addition to the morphometric distances produced by the Q-mode correlations, PCA analysis has also revealed the cranial affinities that exist between our sample of pre-Neolithic skulls and recent Australo-Papuans and Africans.

Cranial morphological affinities between early Africans who lived at the time of dispersal into Eurasia and present-day Africans remain obscure, because informative African samples from the period around 60–50 ky are lacking. Available reports on AMH cranial remains from this period^31–34^ describe archaic or hybrid archaic/modern features, but those remains are fragmentary. Nevertheless, the close affinity between modern Australo-Papuans and modern Africans supports our hypothesis that the first AMH populations to leave Africa successfully were similar in cranial morphology. In this respect, present-day Eurasian people possess a different cranial shape generally attributed to a dry and/or cold climatic adaptation.

A number of different “Out of Africa” models based on skeletal and genomic data have been proposed to explain AMH dispersal. The simplest presents a single dispersal from Africa into Eurasia along migration routes that avoided geographic barriers like the Himalayas^35–38^. Another model proposes multiple waves of migration, with the first dispersal from Africa occurring along the Indian Ocean Rim into Southeast Asia and Australia, followed by a subsequent dispersal to northern Eurasia^38–44^. An initial AMH migration that followed the coastal rim of the Indian sub-continent and continued through Southeast Asia would link well with the later emergence of the archaeologically-defined Hoabinhian stone tool complex in this region.

There is one other point to be emphasized about our findings concerning the Xiaoma cranium from Taiwan. PCA analysis revealed that, in respect to overall cranial size, Xiaoma is very small (PC 1), and comparable to Negrito crania from the Philippines, San from South Africa, and Veddah from Sri Lanka. These people are all known for their short stature and small body size. The Philippine Negrito sample is assumed to belong to our first layer because of its non-metric dental traits, which are highly heritable^29^. Modern Negrito samples included in this study otherwise cluster with second layer (Figs. 4 and 5), but this may reflect long-term heavily interbreeding with surrounding Philippine agriculturalist populations.

Moreover, the small cranium of the Xiaoma individual from Taiwan, together with its apparent short stature from the surviving long bones (a precise estimate of stature is not provided due to incompleteness), supports many Formosan Austronesian oral traditions about the former existence of small and dark-skinned people in Taiwan^24^. Our study not only solves a long-standing mystery in Taiwan society, but also it provides significant insight into the population structure of prehistoric Taiwan, an island that is regarded by linguists as the homeland for the Austronesian language family and many of its speakers^45^.

Our craniometric analysis is the first to be based on female skeletal data from sub-tropical and tropical southern China, Taiwan, and Southeast Asia. The prevailing hot and humid climate offers poor preservation conditions for ancient human burial remains, such that any discoveries can be regarded as precious for anthropological study. Our research indicates the great potential of heretofore overlooked female skeletons for craniometric analysis. The numerous female cranial remains from Neolithic, Bronze, and Iron Age contexts in China and Southeast Asia should also be included in future systematic studies.

## Materials and methods

This study examines female skulls discovered in seven hunter-gatherer sites in southern China, Vietnam, and Taiwan, as listed in Table 1. These female skulls are compared with baseline data observations from ancient Jomon hunter-gatherers (c. 5000–2300 BP) in Japan^46^, and further compared with present-day population samples. The results allow assignment of the samples to their respective layer in the two-layer model. Table 2 presents the cranial measurements used in our study, based on Martin’s definitions^47^.

**Table 2:** Female cranial measurements (mm) of seven sites.

Martin number and measurement	Yahuai	Hang Cho	Mai Da Dieu	Bau Du	Xiaoma	Huiyaotian^a^			Liyupo^b^		
		04HCH3-M1	84MDD-M1	14BD1-M4	No. 5	n	Mean	SD	n	Mean	SD
1. Max. cranial length	190	192	181	172	162	4	195.8	3.5	3	184.3	8.4
5. Basion-nasion length	92	103	89	90		1	98		3	101.3	5.1
8. Max. cranial breadth	147	138	126	133	128	2	135.5	7.8	3	133.3	4.2
9. Min. frontal breadth	109	100	89	93	87	4	96.5	5.8	3	98.8	3.9
10. Max. frontal breadth	130	112	100	110	102	2	114.5	3.5	3	112.3	4.9
12. Max. occipital breadth	114	115		116	101				3	108.5	6.7
17. Basion-bregma height	143	135	(130)	133	(132)^c^	2	145.0	14.1	3	132.3	5.5
29. Frontal chord	120	130	108	110	98	2	108.6	2.3	3	107.1	5.3
30. Parietal chord	119		119	111	107	2	124.1	2.6	2	115.8	7.5
31. Occipital chord	110		91	103		1	100		2	106.9	4.5
40. Basion-prosthion breadth	94			97					3	99.7	4.0
43. Upper facial breadth	115	113	112	108	96	4	110.3	7.8	3	109.9	3.6
45. Bizygomatic breadth	152	128	120	(134)	(138)	3	140.7	1.2	2	131.6	7.6
46. Bimaxillary breadth	98	95	103	110	− 104	3	105.3	3.0	3	103.6	4.4
48. Upper facial height	62	61	58	59	59	5	66.5	4.6	2	62.5	2.2
51. Orbital breadth	(41)	42	38	41	38	3	42.3	3.4	2	41.2	1.1
52. Orbital height	35	31	33	31	31	4	34.8	3.4	2	34.4	0.8
54. Nasal breadth	25	26	27	26	26	4	27.7	1.6	3	27.4	1.4
55. Nasal height	48	47	43	44	43	4	51.2	1.5	2	46.5	2.1
43(1) Frontal chord (FC)	102	106		97	86	2	98.2	5.9	3	104	5.5
43c. Frontal subtense (FS)	14.4	27		14.6		2	9.15	6.6	2	19.2	9.4
57. Simotic chord (SC)		13		10		2	10.4	0.4	2	10.4	0.0
57a. Simotic subtense (SS)						2	2.9	0.0	2	3.9	0.0
46b. Zygomaxillary chord (ZC)	98	97		110	(102)	2	103.6	5.1	2	102.3	8.0
46c. Zygomaxillary subtense (ZS)	17.4	33		18.7		2	19.4	17.5	2	26.2	6.1

Data were collected by Matsumura.

^a^Mean values of six individuals: M7, M19, M20, M32, M39, and M45-2.

^b^Mean values of three individuals: M22, M23, and M28. Values in parentheses are estimated measurements based on reconstructing the missing piece or preserved part.

^c^Estimated measurements based on regression analysis of auricular-basion height (ABH) using 24 Japanese female crania (equation: Basion-bregma height = ABH × 1.05 + 14.26, ABH = 112 mm).

Our study assessed craniometric affinities using Q-mode correlation coefficients based on a dataset of 13 measurements: M1 = maximum cranial length, M8 = maximum cranial breadth, M9 = minimum frontal breadth, M10 = maximum frontal breadth, M17 = basion-bregma height, M43 = upper facial breadth, M45 = bizygomatic breadth, M46 = bimaxillary breadth, M48 = upper facial height, M51 = orbital breadth, M52 = orbital height, M54 = nasal breadth, and M55 = nasal height. These were the most consistently available measurements among the seven selected crania.

The measurements of the comparative cranial samples from modern Eurasian, Melanesian, and African populations, as well as the Jomon sample, were taken from the worldwide database of cranial and dental characteristics compiled by co-author T. Hanihara before 2004. Details of the primary sources used by Hanihara and where samples are stored can be found in his previous publications^38,48,49^. When computing Q-mode correlation coefficients between population samples, the datasets were standardized using the grand means of comparative population samples and standard deviation data from the Jomon sample (Tables S1 and S3).

In order to facilitate the interpretation of phenotypic affinities between samples, we used the Neighbor Net Split method^50^ to the distance (1 − r) matrix of the Q-mode correlation coefficients (r) using SplitsTree, Version 4.05^45^.

Finally, in order to interpret cranial morphometric factors, we conducted principal component analysis (PCA) using the mean value datasets of 13 cranial measurements on all comparative population samples.

### Ethics declarations and research permits

The research permit for each specimen was issued from the associated repository authority. Yahuai (n = 1) and Liyupo (n = 3; M22, M23, M28) are kept in the archaeological storage of Guangxi Institute of Cultural Relic Protection and Archaeology, Nanning, Guangxi, China; Huiyaotian (n = 6; M7, M19, M20, M32, M39, M45-2) are in the Nanning Museum, Nanning, Guangxi, China; Hang Cho (n = 1; 04HCH3-M1) and Mai Da Dieu (n = 1; 84MDD-M1) are in the Institute of Archaeology, Vietnam Academy of Social Science, Hanoi, Vietnam; Bau Du (n = 1; 14BD1-M4) is in the Quang Nam Provincial Museum, Quang Nam, Vietnam; Xiaoma Cave (n = 1; C5) is in the NTU Museum of Anthropology, National Taiwan University, Taipei, Taiwan. The individual (G. Xie, the second author of this study) in the Fig. 3 has given written informed consent to publish these case details.

## Supplementary Information

Supplementary Table S1.

Supplementary Table S2.

Supplementary Table S3.

Supplementary Table S4.

Supplementary Table S5.

Supplementary Table S6.

## Data Availability

All of the raw data used in this study are available in Table 2 and the “Supplementary Files” (Tables S1–S6), available online with this manuscript.
